# Machine learning-based prediction of acute coronary syndrome using only the pre-hospital 12-lead electrocardiogram

**DOI:** 10.1038/s41467-020-17804-2

**Published:** 2020-08-07

**Authors:** Salah Al-Zaiti, Lucas Besomi, Zeineb Bouzid, Ziad Faramand, Stephanie Frisch, Christian Martin-Gill, Richard Gregg, Samir Saba, Clifton Callaway, Ervin Sejdić

**Affiliations:** 1 Department of Acute & Tertiary Care Nursing, University of Pittsburgh, Pittsburgh, PA USA; 2 Department of Emergency Medicine, University of Pittsburgh, Pittsburgh, PA USA; 3 Division of Cardiology, School of Medicine, University of Pittsburgh, Pittsburgh, PA USA; 4 Department of Electrical and Computer Engineering, University of Pittsburgh, Pittsburgh, PA USA; 5 University of Pittsburgh Medical Center (UPMC), Pittsburgh, PA USA; 6 Advanced Algorithms Development Research Center, Philips Healthcare, Andover, MA USA; 7 Department of Biomedical Informatics, University of Pittsburgh, Pittsburgh, PA USA; 8 Department of Intelligent Systems, University of Pittsburgh, Pittsburgh, PA USA

**Keywords:** Acute coronary syndromes, Diagnostic markers

## Abstract

Prompt identification of acute coronary syndrome is a challenge in clinical practice. The 12-lead electrocardiogram (ECG) is readily available during initial patient evaluation, but current rule-based interpretation approaches lack sufficient accuracy. Here we report machine learning-based methods for the prediction of underlying acute myocardial ischemia in patients with chest pain. Using 554 temporal-spatial features of the 12-lead ECG, we train and test multiple classifiers on two independent prospective patient cohorts (n = 1244). While maintaining higher negative predictive value, our final fusion model achieves 52% gain in sensitivity compared to commercial interpretation software and 37% gain in sensitivity compared to experienced clinicians. Such an ultra-early, ECG-based clinical decision support tool, when combined with the judgment of trained emergency personnel, would help to improve clinical outcomes and reduce unnecessary costs in patients with chest pain.

## Abstract (web summary)

Diagnosing a heart attack requires excessive testing and prolonged observation, which frequently requires hospital admission. Here the authors report a machine learning-based system based exclusively on ECG data that can help clinicians identify 37% more heart attacks during initial screening.

## Introduction

Nearly seven million Americans visit the emergency department annually for a chief complaint of chest pain. Approximately ten percent of those patients have an acute disruption in blood supply to the heart attributed to underlying atherosclerotic disease in the coronary arteries, a life-threatening condition referred to as acute coronary syndrome (ACS)^1,2^. However, more than 50–75% of the seven million patients with chest pain are admitted to the hospital because the initial clinical evaluation is not sufficient to rule in or rule out ACS. This problem results from the low sensitivity of the electrocardiogram (ECG) and initial clinical data to predict the presence of ongoing acute myocardial ischemia in those with ACS. As the first available clinical test, the standard 10-s 12-lead ECG can identify a small subset of ACS patients that have ST segment elevation on their ECG, hence the term ST elevation myocardial infarction (STEMI). The majority (>50%) of ACS patients, however, have no such ST elevation on their ECG^3^, and thus require a time-consuming biomarker-driven approach and/or provocative testing to rule in or out acute myocardial ischemia. More sensitive classification tools using the 12-lead ECG could improve speed and accuracy of ACS detection.

Critical narrowing or occlusion of a coronary artery leads to myocardial ischemia in the region supplied by that coronary artery^4,5^. Regional ischemia leads to reduction in the duration, resting potential, and propagation velocity of action potentials in the affected myocardium, which leads to a wide variability in the conduction speeds between various myocardial regions. Variability in conduction speeds between the epicardial and endocardial walls of the affected region results in temporal changes in specific ECG leads facing that region (i.e., features of waveform duration and amplitude in individual leads)^6^, whereas variability in ischemic regions and healthy myocardium results in spatial changes between orthogonal ECG leads (i.e., features of global electrical heterogeneity)^7^. Thus, using both temporal and spatial features of the 12-lead ECG would be more robust in detecting ACS than using temporal waveform features alone, such as ST elevation. We have shown that mild-to-moderate ischemia distorts the temporal–spatial features of the 12-lead ECG before ST changes evolve^8,9^, suggesting that building sensitive ACS classification algorithms using only the 12-lead ECG is plausible.

A single 10-s 12-lead ECG provides a large number of temporal–spatial features and is thus a rich data platform to model and quantify the presence of ongoing myocardial ischemia. Analysis of the high-dimensional, highly correlated ECG features requires sophisticated machine learning (ML) classifiers. A number of ML classifiers to predict ACS using ECG data have been reported in the literature^10–18^. However, most studies either used small and limited public datasets (e.g., MIT-BIH, Physikalisch-Technische Bundesanstalt (PTB), etc.) or used historical ECGs from hospital records in a case–control approach. Other studies focused only on the identification of patients with STEMI^18^, while others used single-lead ECGs or individual heart beats for algorithm development. All of these limitations diminished the clinical utility for predicting acute ischemia in the general, nonselected chest pain populations seen at ED settings. Validation of generalizable ML classifiers on real-world data from prospective cohort studies is needed.

Herein, we present ML-based methods for the prediction of underlying acute myocardial ischemia in patients with chest pain using only the standard 12-lead ECG. We validate and test this approach using two large prospective cohorts from three tertiary care hospitals in the United States of America. Each cohort includes consecutive prehospital 12-lead ECGs obtained during first medical contact. A key feature of our ML method is that it not only utilizes traditional ECG features, but it also takes advantage of novel temporal–spatial features of the 12-lead ECG^19^. Another key element of this method is that feature selection and data recoding is guided by domain-specific knowledge of the pathological nature of acute myocardial ischemia. Using different ML-based classifiers trained and tested on separate prospective cohorts, we arrive at a model that compares to and outperforms clinicians in their expert ECG interpretations based on current practice guidelines.

## Results

### Patient characteristics

The study population consisted of two patient cohorts from the ongoing EMPIRE study (ECG methods for the prompt identification of coronary events)^20^. The first cohort (2013–2014) included 745 patients and the second cohort (2014–2015) included 499 patients with interpretable ECGs (i.e., no excessive noise or artifacts, not in ventricular tachycardia or fibrillation). Paramedics enrolled consecutive patients in both cohorts and acquired 12-lead ECGs in the field prior to any medical treatment. Paramedics transmitted ECGs to our medical command center where they were stored for offline analysis. We collected clinical data for 30 days from the date of index encounter. The primary study outcome used to train classifiers and to test performance was defined as any ACS event. In subsequent sensitivity analyses, we excluded patients with confirmed STEMI on prehospital ECG who were sent to the catheterization lab emergently. Finally, to optimize the clinical utility of our algorithms, we included all ECGs in our analyses, including those with secondary repolarization changes (i.e., pacing, bundle branch block, and left ventricular hypertrophy). Table 1 summarizes the clinical characteristics of each cohort.

**Table 1:** Baseline patient characteristics.

	Cohort 1 (*n* = 745) (training and testing)	Cohort 2 (*n* = 499) (external validation)
Demographics		
Age in years	59 ± 17	59 ± 16
Sex (female)	317 (42%)	243 (49%)
Race (Black)	301 (40%)	202 (40%)
Past medical history		
Hypertension	519 (69%)	329 (66%)
Diabetes mellitus	196 (26%)	132 (26%)
Old myocardial infarction	205 (27%)	122 (24%)
Known CAD	248 (33%)	179 (36%)
Known heart failure	130 (17%)	74 (15%)
Prior PCI/CABG	207 (28%)	124 (25%)
Presenting chief complaint		
Chest pain	665 (89%)	454 (91%)
Shortness of breathing	250 (34%)	234 (47%)
Indigestion, nausea, or vomiting	117 (16%)	109 (22%)
Dizziness or syncope	106 (14%)	79 (16%)
Palpitation	96 (13%)	62 (12%)
Other atypical symptoms	54 (7%)	37 (7%)
Baseline ECG rhythm		
Normal sinus rhythm	648 (87%)	442 (88%)
Atrial fibrillation	71 (9%)	46 (9%)
Pacing	26 (4%)	8 (2%)
Right bundle branch block	31 (4%)	27 (5%)
Left bundle branch block	19 (3%)	16 (3%)
Left ventricular hypertrophy	37 (5%)	24 (5%)
Primary study outcome		
Any ACS event	114 (15.3%)	92 (18.4%)
Prehospital STEMI	31 (4.2%)	18 (3.6%)
NSTE-ACS	83 (11.1%)	74 (14.8%)
Course of hospitalization		
Length of stay (median [IQR])	2.3 [1.0–3.0]	1.2 [0.6-2.5]
Stress testing with SPECT	180 (24%)	115 (23%)
Treated by primary PCI/CABG	74 (10%)	65 (13%)
30-day cardiovascular death	33 (4.4%)	24 (4.8%)

### Dataset derivation and preparation

**Fig. 1:**
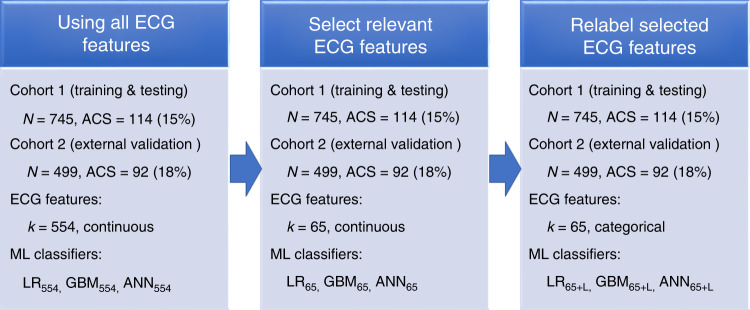
Stages of dataset derivation and preparation prior to developing the ML classifiers. We used all available ECG features (*k* = 554), selected ECG features (*k* = 65), and selected and relabeled ECG features (*k* = 65 + L) to train and test our machine learning (ML) classifiers: logistic regression (LR), gradient boosting machine (GBM), and artificial neural networks (ANN). Cohort 1 was used for training and Cohort 2 was used for independent testing. The primary study outcome was acute coronary syndrome (ACS).

Figure 1 shows the stages of dataset derivation and preparation. First, all ECGs were preprocessed using manufacturer-specific commercial software (Philips Healthcare, Andover, MA) and then we manually inspected tracings for noise and artifact. After ectopic beats were removed and median beats were computed, we extracted 554 temporal–spatial features from each ECG using previously validated, commercial algorithms (Philips Healthcare, Andover, MA). Overall, less than 0.2% of values in all features were missing and these were imputed using either the mean or the mode. We then tested a menu of various ML classifiers and selected for further training and validation the three algorithms that had the best performance: logistic regression (LR), gradient boosting machine (GBM), and artificial neural network (ANN). We further tuned these three classifiers by excluding ECG features that were least likely to have any mechanistic link to the pathogenesis of acute myocardial ischemia, yielding a reduced set of 65 clinically important ECG features. Finally, we recoded the 65 continuous ECG features into categories using previously published cutoff values of clinical significance, for instance, spatial QRS-T angle was relabeled as normal (0–49°), borderline (50–99°), and abnormal (≥100°). There were a total of nine classifiers trained and tested in this paper.

### Classification performance using various ECG feature subsets

**Fig. 2:**
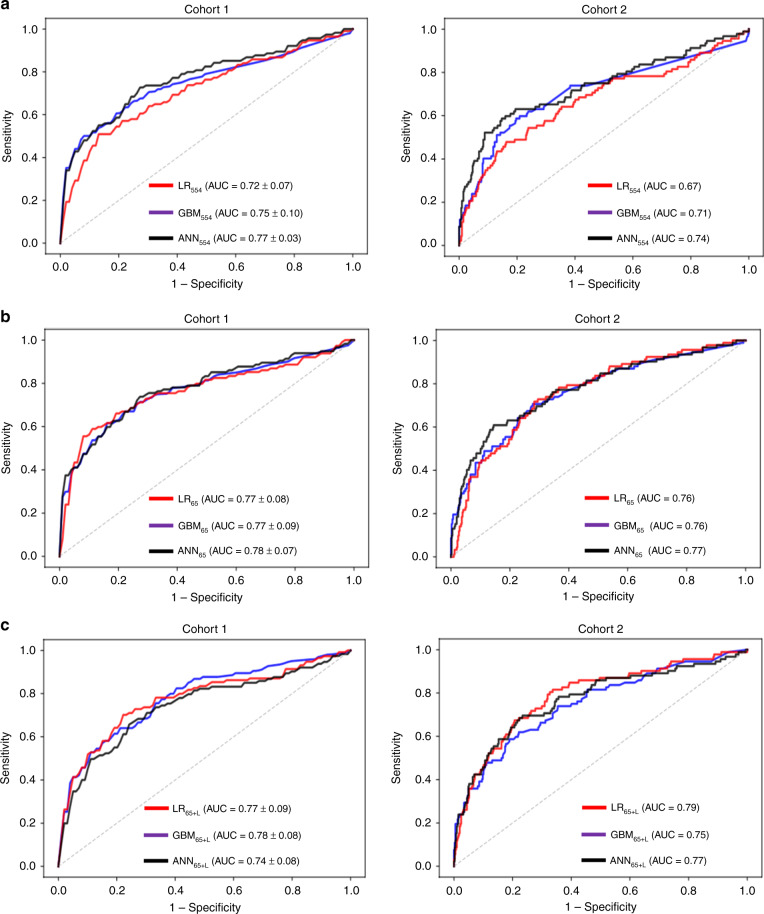
Classification performance using machine learning classifiers. This figure shows the ROC curves of logistic regression (LR), gradient boosting machine (GBM), and artificial neural network (ANN) classifiers using **a** all available ECG features (*k* = 554), **b** selected ECG features (*k* = 65), and **c** selected and relabeled ECG features (*k* = 65 + L). Cohort 1 was used for training with tenfold cross-validation, the figure shows mean ROC curve with ±2 standard errors. Cohort 2 was used for independent testing using the algorithm trained on Cohort 1.

Using tenfold cross-validation approach, we first trained each ML classifier using all 554 features (LR_554_, GBM_554_, and ANN_554_) on Cohort 1, and then tested performance on Cohort 2. Figure 2a shows the ROC curves for each classifier. Although GBM_554_ and ANN_554_ outperformed LR_554_ using all available ECG features, we observed a wide variability in classifiers’ performance with poor generalizability to testing set, reflecting low bias–high variance tradeoff.

Next, we trained and tested the performance of each ML classifier using only the 65 ECG features deemed as clinically relevant to the pathogenesis of acute myocardial ischemia (LR_65_, GBM_65_, and ANN_65_). Figure 2b shows the ROC curves for these classifiers. Interestingly, all three classifiers performed equally on the training set and they generalized well on the independent testing set, reflecting low bias–low variance tradeoff.

Finally, we trained the three classifiers using the same 65 features after they were relabeled using previously validated cutoff thresholds of clinical significance (LR_65+L_, GBM_65+L_, and ANN_65+L_). Figure 2c shows the ROC curves for these classifiers. Although these classifiers performed well on the training set, we again observed a wide variability in classifiers’ performance with poor generalizability to testing set, reflecting low bias–low variance tradeoff.

### Comparing ML classifiers to reference standard

**Fig. 3:**
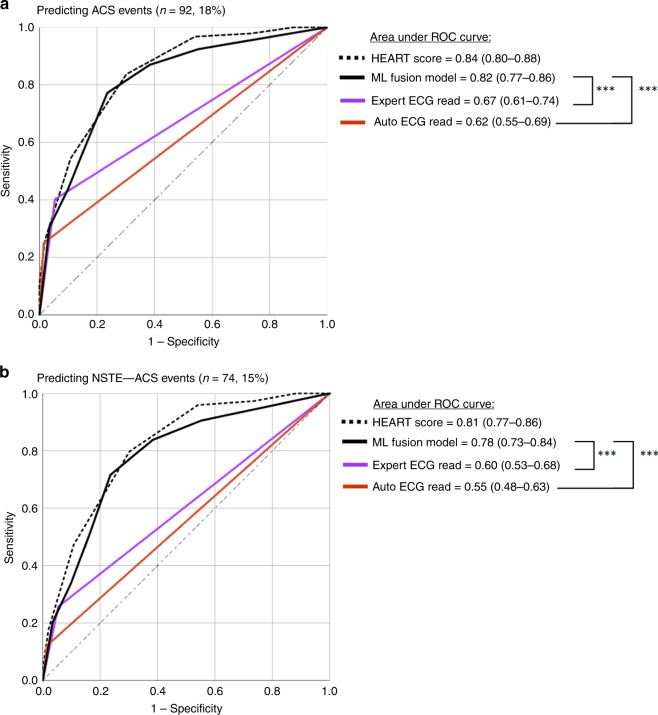
Classification performance of final model on testing set (*n=* 499). This figure compares the area under ROC curve (95% confidence interval) between our machine learning (ML) fusion model against experienced clinicians and against rule-based commercial interpretation software for detecting **a** any acute coronary syndrome (ACS) event, and **b** non-ST elevation acute coronary syndrome events (NSTE-ACS). ***p < 0.001 using two-sided DeLong’s nonparametric approach.

We used the ML classifiers with best low bias–low variance tradeoff to create a simple fusion model. This fusion model was based on vote count of classifiers built on the reduced (LR_65_, GBM_65_, and ANN_65_) and labeled (LR_65+L_, GBM_65+L_, and ANN_65+L_) datasets. We used three or more votes as the cutoff to compute diagnostic performance metrics for this model as compared with two current ECG reference standards: (1) expert ECG read by clinicians and (2) automated ECG reads by commercial rule-based software. To get these annotations, each 12-lead ECG was annotated according to the fourth Universal Definition of Myocardial Infarction consensus statement^21^ by two experienced clinicians who were blinded from study outcome. We used Philips diagnostic 12/16 lead ECG analysis program (Philips Healthcare, Andover, MA) for automated ECG read. Figure 3 and Table 2 show the ROC curves and diagnostic accuracy metrics for the ML fusion model against the two ECG reference standards. To place these comparisons in a context, we show the classification performance of the history, ECG, age, risk factors, troponin (HEART) score obtained at the ED based on all available clinical, laboratory, and ECG data.

**Table 2:** Diagnostic accuracy measures on testing set (*n* = 499).

Predicting any ACS event			
	Ml fusion model	Expert ECG read	Automated ECG read
Sensitivity	0.77 (0.67–0.85)	0.40 (0.30–0.51)	0.25 (0.17–0.35)
Specificity	0.76 (0.72–0.81)	0.94 (0.92–0.96)	0.98 (0.97–0.99)
PPV	0.43 (0.38–0.48)	0.63 (0.51–0.73)	0.79 (0.62–0.90)
NPV	0.94 (0.91–0.96)	0.87 (0.86–0.89)	0.85 (0.82–0.88)
Predicting NSTE-ACS events			
Sensitivity	0.72 (0.60–0.81)	0.26 (0.16–0.37)	0.12 (0.06–0.22)
Specificity	0.76 (0.72–0.80)	0.94 (0.92–0.96)	0.98 (0.97–0.99)
PPV	0.36 (0.31–0.41)	0.46 (0.33–0.60)	0.60 (0.35–0.80)
NPV	0.94 (0.91–0.93)	0.87 (0.85–0.89)	0.86 (0.85–0.87)

PPV, positive predictive value; NPV, negative predictive value.

Figure 3a demonstrates that ML classifiers outperform expert clinicians and commercial ECG algorithms in detecting ACS events. While maintaining higher negative predictive value (NPV), our ML fusion model demonstrates 37% gain in sensitivity compared with experienced clinicians and 52% gain compared with commercial ECG algorithms (Table 2), corresponding to a net reclassification improvement (NRI) of 0.19 (95% CI 0.06–0.31) and 0.30 (95% CI 0.19–0.41), respectively. Furthermore, supplementing our ML algorithm with important patient history data typically available during first medical contact did not result in any additional improvement in classification performance.

Next, to explore the performance of our model in detecting non-ST elevation ACS (NSTE-ACS) events, we removed the prehospital STEMI cases and repeated our analyses (Fig. 3b). As seen in this figure, our ML fusion model still outperforms experienced clinicians and commercial ECG software in detecting the majority of ACS events (Table 2), with NRI of 0.28 (95% CI 0.13–0.43) against experienced clinicians and 0.37 (95% CI 0.26–0.49) against commercial algorithms. This finding supports the notion that unlike current ECG reference standards that are heavily geared toward evaluating ST amplitude changes, our ML algorithm takes into account the subtle temporal–spatial signatures of ischemia, which explains the large gain we observed in the diagnostic accuracy.

Finally, to identify potential room for improvement, we interrogated the sources of false negatives in the classifications of our ML model. On independent testing, there were 21 patients with ACS misclassified as no disease. We investigated the ECGs of these cases and identified the following potential sources of error: excessive baseline wander (*n* = 6), frequent PVCs (*n* = 3), tachycardia > 100 bpm (*n* = 2), and left ventricular hypertrophy (*n* = 2).

## Discussion

This study built and tested a classification algorithm that uses available ECG data from first medical contact to predict ACS in consecutive, unselected patients presenting to ED with chest pain. Using different ML-based classifiers trained and tested on separate prospective cohorts, we arrived at a generalizable model that outperforms both commercial interpretation software as well as experienced clinicians. Our findings show that, by incorporating existing clinical knowledge in classification decisions, linear prediction models like LR can be equivalent to complex and computationally expensive algorithms like ANN and GBM. On the independent test set, our final ML fusion classifier, while maintaining higher NPV, achieved 39% gain in sensitivity compared with commercial interpretation algorithms and 24% gain in sensitivity compared with experienced clinicians. To our knowledge, this is the first clinical study that prospectively validated and tested the performance of ML-based models on two separate cohorts to predict ACS using only the prehospital 12-lead ECG.

Our findings have several important clinical implications. First, using an ultra-early, ECG-based clinical decision support tool, when combined with the judgment of trained emergency personnel, could be imperative for improving outcomes in patients with chest pain. Our gain in net classification improvement implies that 37–59% of patients with ACS could be better targeted for transfer to appropriate destinations (e.g., centers with advanced cardiac care units or PCI capabilities) or for the initiation of guideline-recommended anti-ischemic therapies. Our high NPV suggest that our algorithm could also be useful for the early and safe discharge of chest pain patients at low risk of ACS. This could save substantial time and cost compared with traditional chest pain evaluations reliant on biomarkers and provocative testing performed over a 24-h hospital observation. Second, strength of our predictive model lies in its real-time applicability and scalability since it could be automated and directly integrated into existing ECG machines without the need to input additional clinical data into the model. This means that our model can be very useful in non-tertiary care settings where more invasive diagnostics might not be readily available. Third, the real-time clinical decision support that could be provided by our model is specifically useful to nonspecialists and nurses or prehospital personnel with limited experience in ECG interpretation. The classification performance of our model not only outperformed rule-based predictions by standard commercial software, but also met and outperformed the expert ECG interpretation by trained physicians. This means that our algorithm can be used by nonspecialized emergency personnel to screen patients and identify the subset of patients whose ECGs need to be further evaluated by offsite experts, a strategy that has long been shown to improve outcomes in those with confirmed ACS^22^. Finally, our algorithm can be used to detect ACS in patients whose ECG is confounded by baseline abnormalities such as pacing and bundle branch blocks. Current clinical sensitivity in classifying these patients is low, and our algorithm’s ability to triage these vulnerable patients would significantly enhance the generalizability of our approach to real-world clinical settings.

Applying ML algorithms to predict ACS has been widely described in literature. A challenge in the development of such models using ECG data is the absence of relevant datasets for training and validation. Most prior algorithms^23–34^ have used the open-source PTB diagnostic ECG database. This highly selected dataset contains the 12-lead ECGs of only 200 subjects (148 ACS and 52 healthy controls). Although most of these ML classifiers report accuracy that ranges from 93.5 to 98.8%, the generalizability of such models to real-world clinical settings remain questionable, and it is likely these algorithm were overfitting the data contained in the PTB dataset

On the other hand, there were few studies that used clinical datasets to build ML classifiers. However, most of these studies combined classical ECG features (e.g., diagnostic ST–T amplitude changes) with a full range of other clinical data elements (e.g., patient history, physical exam abnormalities, laboratory values, and/or diagnostic tests)^10,15,17,35^. Despite the high accuracy achieved by these models (≥0.90), classifiers that incorporate such extensive findings from patient clinical profiles have limited utility during early patient triage decisions.

There are two prior studies that used only ECG data to build ML-based classifiers. Forberg et al.^36^ trained and cross-validated ANN and LR classifiers on a dataset of 861 patients with chest pain (ACS = 344, 40%). Using 228 ECG features (i.e., 19 temporal measures from each of the 12 leads), their classifiers achieved AUC of 0.86 and 0.88, respectively, providing nearly 13% gain in sensitivity compared with expert clinicians and 20% gain compared with automated interpretation software. Although LR classifier developed by Forberg et al. (AUC = 0.88) performed better compared with ours (AUC = 0.79), it is worth noting that their approach selectively recruited positive cases to enrich their dataset for ACS, making their classes artificially better balanced compared with ours (ACS prevalence = 40% vs. 18%). In addition, their classifier was not evaluated on an independent test set, suggesting that their classifier yielded a too optimistic estimate of the ROC performance. Another clinical study that is relevant to our current work is the study by Green et al.^13^ that trained ANN and LR classifiers on 643 consecutive patients with chest pain (ACS = 130, 20%). Using a set of 16 ECG features (i.e., duration, amplitude, area, and slope measures of QRS and ST segment) that were reduced using PCA from nearly 72 features, their classifiers achieved an AUC of 0.80 and 0.71, respectively. However, these results were not evaluated on an independent test set, again raising concerns about the generalizability of their model.

How to select the most appropriate ML approach in clinical applications is a debatable issue. Prior studies have generally showed that ANN outperforms LR classifiers in the task of ACS prediction. Green et al.^13^ specifically compared the performance of ANN against LR, taking into account computational data reduction using PCA, and found that the earlier provides significant clinical advantage in risk stratification. Our data supported this notion that nonlinear models like ANN and GBM are more powerful tools to handle the high-dimensional, highly correlated nature of excessive ECG features. However, our data interestingly showed that feature selection and annotation based on existing clinical knowledge can boost the classification performance of linear models like LR. This is reasonable given that data reduction and labeling could reduce the dimensionality and complexity in the data. Although this significant improvement in LR classifier is yet to be compared against other data reduction techniques in subsequent methodological studies, it has important technical implications. First, if it is confirmed that simple linear classifiers can be equivalent to complex nonlinear models such as ANN, then future applications can focus on less computationally exhaustive models like LR classifiers. Second, and more importantly, LR has the appealing property of being fully interpretable by clinicians, which can improve the clinical utility by removing the black-box stigma of ML. Identifying a subset of features that are prevailing in the prediction of ACS can shed the light on some hidden important disease pathways in our current understanding of the electrocardiographic presentation of acute myocardial ischemia.

Our study has several strengths that addressed some of the existing gaps in the literature. First, unlike previous studies, we did not exclude ECGs confounded by baseline abnormalities such as pacing and bundle branch blocks, which would significantly enhance the generalizability of our approach to real-world clinical settings. This was evident by the performance of our model on the external validation prospective cohort that had ACS prevalence similar to what is seen in real-world patient populations. Second, our dataset was unique in that it used the prehospital 12-lead ECG rather than the initial ECG from the emergency department. Both have been previously shown to be dissimilar^37^; the prehospital ECG could capture the subtle and transient acute cardiac ischemia during its ongoing evolution, changes which could be easily masked on ECGs obtained at the emergency department following early treatment. To our knowledge, this is the first study to build a generalizable ML classifier to predict the likelihood of ACS using only the prehospital ECG data.

Nevertheless, our study had some limitations. First, the number of the abstracted ECG features was not proportional to the size of the dataset, which might have affected the performance of our classifiers. Increasing the number of patients would probably lead to increased performance. Second, although manual feature selection had a positive effect on the performance of our ML classifiers, further data-driven techniques for feature selection need to be further investigated. Third, the majority of commercial ECG software are designed based on strict criteria geared toward rule in STEMI, which explain the very low sensitivity for NSTE-ACS detection observed in this study. As such, the emphasis of our approach should focus on comparing the performance of our ML classifiers against experienced clinicians. Finally, although not widely used in the USA, high sensitivity cardiac troponin I has been shown as an indispensable rule out tool in patients with suspected ACS in the emergency department^38^. On contrast, our algorithm has been shown to improve the net gain classification performance of patients with ACS (rule in), plus it can aid decisions at the prehospital setting. A recent study has shown that point-of-care troponin assays has a sensitivity of only 27% when used during ambulance transport^39^.

In conclusion, using features extracted from only the prehospital 12-lead ECG, we arrived at a generalizable ML model that outperforms both commercial interpretation software and experienced clinician interpretation. Such ultra-early, ECG-based clinical decision support tool, when combined with the judgment of trained emergency personnel, could be imperative for improving clinical outcomes and reducing unnecessary costs in patients with chest pain. Furthermore, domain-specific knowledge can boost the classification performance of linear models like LR, which has important implications for building user-friendly and acceptable decision support tools for wider clinical use.

## Methods

### Design and settings

The dataset used in this paper was obtained from the EMPIRE study. EMPIRE is a prospective observational cohort study that recruited consecutive, nontraumatic chest pain patients transported by emergency medical services to one of three UPMC-affiliated tertiary care hospitals (UPMC Presbyterian, Mercy, and Shadyside). As per prehospital medical protocols, standard 10-s 12-lead ECGs are obtained on all patients with suspected ACS during first medical contact. If the initial patient evaluation by paramedics was judged to be highly suspicious for cardiac ischemia, then the ECG was transmitted to UPMC medical command, where the raw digital ECG data are permanently stored. In the EMPIRE study, we recruited all consecutive chest pain patients with transmitted ECG data.

This study was approved by the Institutional Review Board of University of Pittsburgh, and all relevant ethical regulations on human experiments, including the declaration of Helsinki, have been followed. This study had minimal patient risk, it was observational, there was no change to routine medical care, there was no direct contact with patients, and follow up data were collected after all routine medical care was completed. Thus, and in order to recruit an unbiased and representative cohort of consecutive patients, data were collected under a waiver of informed consent. The only minimal risk was breach of confidentiality during data abstraction from the electronic health record. As such, only authors who had immediate clinical responsibilities toward the study population (C.M.-G. and C.C.), or their delegates (Z.F. and S.F.), had access to identifiable health information and study ID linkage list. All other authors had access to only de-identified data during data analysis.

The current dataset consisted of 1251 patients from the EMPIRE study from which 30-day follow up outcome data were available. To estimate the minimum sample size required for adequate AUC analysis of new diagnostic tests, we used the methods described by Hajian-Tilaki^40^. So that the maximum marginal error of estimates (precision) does not exceed 5% with 95% confidence level, at desired validation values of sensitivity and specificity of 90%, the minimum sample size required for ACS detection given a prevalence of at least 15% is 927. Moreover, given that ML does not follow the same statistical rules for sample size estimation, we assessed the adequacy of sample size for our ML classifiers by evaluating models for overfitting (common with inadequate sample size). In our analysis, our algorithms generalized well from Cohort 1 to Cohort 2, suggesting that data were not overfitted and the sample size was adequate.

### Study outcome

The primary outcome of the study was the presence of ACS (myocardial infarction or unstable angina) during the primary indexed admission, defined according to the 4th Universal Definition of MI guidelines as the presence of symptoms of ischemia (i.e., diffuse discomfort in the chest, upper extremity, jaw, or epigastric area for more than 20 min) and at least one of the following criteria: (1) elevation of cardiac troponin I (>99th percentile) with or without subsequent development of diagnostic ischemic ECG changes during hospitalization, (2) imaging evidence of new loss of viable myocardium or new regional wall motion abnormalities, or (3) coronary angiography or nuclear imaging demonstrating >70% stenosis of a major coronary artery with or without treatment^21^. Two independent reviewers annotated available medical data and adjudicated this outcome based on serial ECGs, results of cardiac diagnostic tests (e.g., echocardiography, angiography, biomarkers lab test) and other pertinent information (e.g., past medical record, prescribed medications). Patients discharged from the emergency department were classified as negative for ACS if they had no 30-day adverse events. Disagreements were resolved by a third reviewer. Finally, in subsequent sensitivity analyses, we tested the performance of our algorithms in detecting patients with NSTE-ACS after excluding patients with confirmed STEMI on their prehospital ECG and who were sent to the catheterization lab emergently.

### ECG reference standard

Two independent physicians who were blinded from the study outcome evaluated the 12-lead ECG image of each patient. All ECGs were de-identified, labeled with study ID, and were stored on a secure server. First, ECGs with excessive noise or ventricular tachycardia or fibrillation were excluded from this analysis (Cohort 1 *n* = 5/750; Cohort 2 *n* = 2/501). All other available ECGs, including those with pacing, bundle branch blocks, atrial fibrillation, or left ventricular hypertrophy, were included in the analysis. Second, each reviewer labeled diagnostic ECG changes according to the fourth Universal Definition of Myocardial Infarction consensus statement^21^ as two contiguous leads with (1) ST elevation in V2–V3 ≥ 2 mm in men ≥ 40 years, ≥2.5 mm in men < 40 years, or ≥ 1.5 mm in women; or ST elevation ≥  1 mm in other leads; (2) new horizontal or downsloping ST depression ≥ 0.5 mm; or (3) T-wave inversion > 1 mm in leads with prominent R wave or R/S ratio > 1. Finally, taking into account the prior criteria for ST–T changes and all other ECG findings suspicious for ischemia (i.e., contiguous territorial involvement, evidence of reciprocal changes, changes beyond those caused by secondary repolarization, and lack of ECG evidence of nonischemic chest pain etiologies), each reviewer made a final determination about the likelihood of underlying ACS (yes/no). Disagreements were resolved by a board-certified cardiologist.

Furthermore, to place the comparisons between our ML classifiers and ECG reference standards in a context, we used the HEART score obtained at the ED as a reference to the classification performance achieved in current clinical practice. We computed HEART score as described in details elsewhere^41^.

### ECG data

All digital ECG files were acquired using HeartStart MRX monitor-defibrillator at 500 samples/second (Philips Healthcare). Standard ECG signal preprocessing was completed using manufacture-specific commercial software at the Philips Healthcare Advanced Algorithm Research Center (Andover, MA). The raw digital ECG signals were first decompressed and the ECG leads were extracted. Noise, artifact, and ectopic beats were removed, and the representative average beat for each ECG lead was computed to eliminate residual baseline noise and artifacts, yielding a high signal-to-noise ratio and stable average waveform signal for each of the 12 leads. Feature extraction was performed on these representative beats.

First, from each of the 12 leads, the amplitude, duration, and/or area measures of the P wave, Q wave, R wave, S wave, qR wave, rS wave, QRS complex, QRS peak, ST segment, T wave, STT wave, QT interval, PP interval, RP interval, and SP interval were computed (*k* = 384). In addition, the amplitude of ST onset, ST peak, ST offset, and J+80, as well as ST slope were computed from each lead (*k* = 60). This yielded a total of 444 temporal ECG features. Then, all representative beats were aligned and a set of global measures were obtained. Extracted features included QRS, JT_end_, J_Tpeak_, T_peak-end_, and QT interval measures (*k* = 6); QRS and T axes from the frontal, horizontal, and XYZ planes (*k* = 16); spatial angle between QRS and T waveforms (*k* = 6); inflection, amplitude, and slope of global QT, QRS, and T wave in frontal and horizontal planes (*k* = 56); ratios between PCA eigenvalues of QRS, STT, J, and T subintervals (*k* = 13); T-wave morphology and loop (*k* = 7); signal noise values (*k* = 6); regional MI scar using Selvester score (*k* = 19); and injury vector gradient and amplitude (*k* = 14). This yielded a total of 143 spatial ECG features. All extracted ECG features were then *z*-score normalized.

We had a total of 587 temporal–spatial ECG features extracted from each 12-lead ECG. First, to safeguard against systematically missing data due to noise and artifact, which are usually common in prehospital setting (e.g., unsticking of electrodes), we manually evaluated each record to exclude ECGs of poor quality or with failed leads. We found that a very small subset of patients had uninterpretable ECGs due to excessive noise (<3%). We speculate that this low rate is because we enrolled only patients with ECG transmitted to medical command center. Paramedics routinely repeat poor or problematic ECGs in the field before they transmit to a command physician for medical consultations. After excluding these poor ECGs from further analysis, subsequent dataset preparation identified a subset of features (*n* = 33) that were completed unbalanced (i.e., <5% with nonzero values). Upon further evaluation by clinical experts, we found out that zero values were the normal variant on most of these ECG features. For instance, there are usually no S waves in leads II, aVL, V5, and V6, and most leads have no Q waves, which means it is acceptable to see “zero” values for these features. After removing these features, the final dataset included 554 features available for training ML classifiers. Any residual missing values at random were imputed using the mean or mode.

**Fig. 4:**
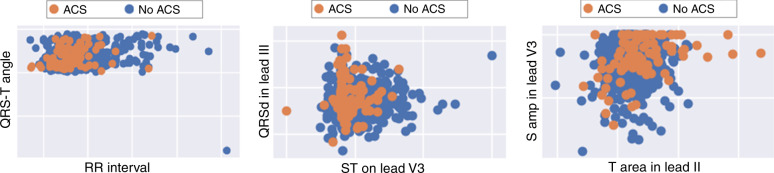
Scatterplot matrix of some selected features using recursive feature elimination. This figure shows selected ECG features with the two-dimensional display scatterplot matrices. These plots show how linear correlations fail to separate patients with or without acute coronary syndrome (ACS), which explains why nonlinear classifiers were computationally favored over linear classifiers in our study.

Next, to evaluate the complexity of the nonlinear correlations in ECG features evaluated, we used recursive feature elimination technique to identify the most important features nested in the developed ML classifiers. Figure 4 shows some selected features with the two-dimensional display scatterplot matrices. As expected, linear correlations failed to separate patients with or without the disease, computationally favoring nonlinear classifiers like GBM and ANN over linear classifiers like LR. As such, two expert clinician scientists reviewed the important ECG features and identified the features that were clinically relevant to the pathogenesis of myocardial ischemia. The following 65 features were identified: (1) amplitude of J+80 and T wave from each of the 12 leads (*k* = 24); (2) QRS, JT_end_, J_Tpeak_, T_peak-end_, and QT interval measures (*k* = 6); (3) QRS and T axis in the frontal plane (*k* = 2); (4) spatial angle between QRS and T waveforms (*k* = 6); (5) inflection, amplitude, and slope of T wave in frontal plane (*k* = 5); (6) ratios between PCA eigenvalues of QRS, STT, J, and T subintervals (*k* = 13); (7) T-wave morphology and loop (*k* = 7); and signal noise values (*k* = 2).

Finally, we annotated this reduced subset of 65 features to denote normal vs. abnormal thresholds based on published cutoff clinical values. Symbolic dynamics is a mathematical modeling approach that aims to convert infinite dynamical systems into discrete intervals each of which denotes a particular state, with the discrete labels (dynamics) given by the shift operator. This step seemed necessary given that many ECG features are nonlinear. For example, both a T-wave amplitude < 0 or >1 mV usually indicate a change of state (i.e., myocardial ischemia) along the continuum of T-wave amplitude value. This clinically annotated and reduced subset was used during the final stage of model development.

### Clinical data

Although the ECG is the only diagnostic tool available during early triage, age and sex are important predictors of ACS during first medical contact. In fact, ECG data are always age and sex normalized, that is users have to enter these values into the ECG machine before ECG acquisition; and these entries are used in the machine-provided, rule-based interpretations. The guideline recommendations used by clinicians were age- and sex-specific as well^21^. Given that we aimed to compare our ML classifiers with the reference standard by expert clinicians and available commercial software, we therefore decided to keep the age and sex as input features in all of our ML classifiers as we tuned our models.

### ML classifiers

All analyses were performed using Matlab R2013a. We performed supervised learning using a menu of different ML classifiers and selected for further training and validation the algorithms that had the best performance: LR, GBM, and ANN. Other ML algorithms explored but did not seem to perform as the selected ones include: SVM, Naive Bayes, random forest, etc. Regardless of the tuning of their parameters, these latter methods performed poorly and were therefore excluded from the study.

The selected classifiers provide complementary values for predicting and classifying dire outcomes in clinical research. LR classifiers simplify the relationship between the input and the output, but becomes computationally expensive and limited when the relationship in the model is complex. In contrast, GBM builds an efficient classifier by using regression decision trees as weak learners, and combining them into a single stronger learner. ANN models complex relationships between the input and the output, thanks to its hidden layers, the activation functions of its neurons, and the back propagation method for updating its unit weights.

The ANN implemented in this study had one hidden layer with a number of hidden units adjusted to each version of the dataset. The network used the rectified linear unit activation function for the forward propagation and the Adam solver to perform the back propagation and update the units’ weights. These parameters were determined via a grid search, trying to optimize the mean AUC on the test splits of the tenfold cross-validation. In the same way, the parameters of LR and GBM were determined using the same grid search optimization approach. The LR model used limited-memory BFGS as optimization solver, the value of the regularization parameter was adjusted on each version of the dataset, and dedicated weights were attributed to each sample in order to account for the class imbalance. The GBM model used the deviance as loss function, and the values of the learning rate and the number of boosting iterations were adjusted on each version of the dataset.

To train and validate the models and account for the imbalance of the output classes, the training data (*n* = 745) was split into stratified tenfold cross-validation sets; hence, the classes were equally distributed from one split to the other. We then tested the performance of each classifier on the independent test set (*n* = 499). The model development and testing went into three stages: (1) Using all extracted ECG features as input (LR_554_, GBM_554_, and ANN_554_); (2) Using only clinically relevant EC features as input (LR_65_, GBM_65_, and ANN_65_); and (3) Using the clinically relevant ECG features labeled according to published cutoff standards as input (LR_554+L_, GBM_554+L_, and ANN_554+L_). Finally, we selected the six classifiers with the best low bias–low variance tradeoff and created a simple hybrid/fusion model based on vote count. Classifications assigned by each model on the test set (disease vs. no disease) were used as votes. We considered three or more votes as the threshold to compute diagnostic performance values for this fusion model.

### Algorithm performance and statistical testing

The different performance metrics we refer to in this paper are the AUC, sensitivity, specificity, and positive predictive value and NPV evaluated for a certain classification cutoff value. We used tenfold cross-validation in Cohort 1 to estimate the statistical uncertainty around ROC curves. To incorporate the uncertainty in the selection of cutoffs, we first calculated the mean ROC of the tenfolds, then selected the ROC coordinate point that maximized the sensitivity and kept the specificity above the arbitrary minimum level of 70%. This selection aimed toward creating a good rule in model but with acceptable specificity. The ROC cutoff value corresponding to this optimum was thus determined for each classifier studied on the training set (Cohort 1), and the corresponding diagnostic metrics were computed on the test set (Cohort 2). This independent testing supported whether or not the trained models behaved consistently on new unseen patients. Lastly, we compared the performance of our final ML classifier against standard reference on the test set. We used DeLong’s nonparametric approach to compare two ROC curves derived from related sample, with a *p* value of <0.05 (two-sided) indicating a significant difference between two diagnostic tests^42^. We then computed the NRI for our ML algorithm against the standard reference using previously published methods^43^.

### Reporting summary

Further information on research design is available in the Nature Research Reporting Summary linked to this article.

## Supplementary information

Reporting Summary

## Data Availability

The data that support the findings of this study are available on request from the corresponding author S.S. The data are not publicly available due to intellectual property claims under U.S. Patent and Trademark Office. However, source data for Fig. 3 and Table 2 are provided with the paper. Source Data are provided with this paper.

## Source data

Source Data

## Code availability

The custom MATLAB code used in this study is available from the corresponding author upon reasonable request. This code is copyrighted by University of Pittsburgh and is to be used only for educational and research purposes. Any commercial use, including the distribution, sale, lease, license, or other transfer of the code to a third party, is prohibited. For inquiries regarding commercial use of the code, please contact University of Pittsburgh’s Innovation Institute, Office of Technology Management. Source Data are provided with this paper.
