# Supplementary material for: Machine learning-based prediction of acute coronary syndrome using only the pre-hospital 12-lead electrocardiogram
Source: Nat Commun. 2020 Aug 7;11:3966. doi: 10.1038/s41467-020-17804-2 (PMC7414145; doi:10.1038/s41467-020-17804-2)
Supplement: Supplementary file 1 — Reporting Summary [file 41467_2020_17804_MOESM1_ESM.pdf]

nature research

Corresponding author(s): Al-Zaiti, Salah S.

Last updated by author(s): 10/16/2019

Reporting Summary

Nature Research wishes to improve the reproducibility of the work that we publish. This form provides structure for consistency and transparency in reporting. For further information on Nature Research policies, see Authors & Referees and the Editorial Policy Checklist .

Statistics

For all statistical analyses, confirm that the following items are present in the figure legend, table legend, main text, or Methods section.

n/a	Confirmed	
☐	☒	The exact sample size (*n*) for each experimental group/condition, given as a discrete number and unit of measurement
☐	☒	A statement on whether measurements were taken from distinct samples or whether the same sample was measured repeatedly
☐	☒	The statistical test(s) used AND whether they are one- or two-sided<br>*Only common tests should be described solely by name; describe more complex techniques in the Methods section.*
☐	☒	A description of all covariates tested
☐	☒	A description of any assumptions or corrections, such as tests of normality and adjustment for multiple comparisons
☐	☒	A full description of the statistical parameters including central tendency (e.g. means) or other basic estimates (e.g. regression coefficient) AND variation (e.g. standard deviation) or associated estimates of uncertainty (e.g. confidence intervals)
☐	☒	For null hypothesis testing, the test statistic (e.g. *F*, *t*, *r*) with confidence intervals, effect sizes, degrees of freedom and *P* value noted<br>*Give P values as exact values whenever suitable.*
☒	☐	For Bayesian analysis, information on the choice of priors and Markov chain Monte Carlo settings
☒	☐	For hierarchical and complex designs, identification of the appropriate level for tests and full reporting of outcomes
☐	☒	Estimates of effect sizes (e.g. Cohen's *d*, Pearson's *r*), indicating how they were calculated

Our web collection on statistics for biologists contains articles on many of the points above.

Software and code

Policy information about availability of computer code

Data collection	No software used
Data analysis	Matlab R2013a

For manuscripts utilizing custom algorithms or software that are central to the research but not yet described in published literature, software must be made available to editors/reviewers. We strongly encourage code deposition in a community repository (e.g. GitHub). See the Nature Research guidelines for submitting code & software for further information.

Data

Policy information about availability of data

All manuscripts must include a data availability statement. This statement should provide the following information, where applicable:

- Accession codes, unique identifiers, or web links for publicly available datasets
- A list of figures that have associated raw data
- A description of any restrictions on data availability

The data that support the findings of this study are available on request from the corresponding author S.S.A. The data are not publicly available due to intellectual property claims under U.S. Patent and Trademark Office. However, source data for figure 3 and Table 2 are provided with the paper.

Field-specific reporting

Please select the one below that is the best fit for your research. If you are not sure, read the appropriate sections before making your selection.

☒ Life sciences ☐ Behavioural & social sciences ☐ Ecological, evolutionary & environmental sciences

Life sciences study design

All studies must disclose on these points even when the disclosure is negative.

Sample size	To estimate the minimum sample size required for adequate AUC analysis of new diagnostic tests, we have used the equations described in Journal of Biomedical Informatics. 2014;48:193-204. So that the maximum marginal error of estimates (precision) does not exceed 5% with 95% confidence level, at desired validation values of SE/SP of 90%, the minimum sample size required for ACS detection given a prevalence of at least 13% is 1,070. Our sample size in this paper was 1,250, and after excluding uninterpretable cases we had n of 1,066. Nevertheless, we used machine-learning (ML) algorithms to perform the predictions. ML does not follow the same statistical rules for sample size estimation, and it can be effective with datasets as small as 200 patients. A good approach to assess the adequacy of sample size for ML is to evaluate model for overfitting (common with inadequate sample size). In our analysis, all three ML algorithms generalized from Cohort 1 to Cohort 2, suggesting that data were not overfitted and the sample size was adequate.
Data exclusions	We excluded patients with uninterpretable ECGs (i.e., excessive noise or artifacts), and those with secondary repolarization confounders (i.e., pacing, bundle branch block, left ventricular hypertrophy, ventricular arrhythmias). These confounders lead to imprecise computation of predictive features and were therefore excluded.
Replication	ECG features extraction was performed using manufacture-specific commercial software at the Philips Healthcare Advanced Algorithm Research Center (Andover, MA). This is the state-of-the-art approach that would provide calculations similar to those used clinically. In addition, to safeguard against imprecise calculations due to noise and artifact, which are usually common in prehospital setting (e.g., unsticking of electrodes), we manually evaluated each record to exclude ECGs of poor quality or with failed leads.
Randomization	This was an observational study and there was no randomization. To avoid any selection bias, we enrolled ALL consecutive patients calling 9-1-for chest pain in the City of Pittsburgh during the study period.
Blinding	This was an observational study and there was no blinding of random allocation. However, we used blinding of outcome / predictor data to ensure quality: 1) clinicians adjudicating the clinical outcomes were blinded from prehospital ECG data; 2) researchers at Philips had only access to the ECG data to extract features and did not have any clinical data; 3) data scientists completing the machine learning algorithms only had the outcome data for Cohort 1; and 4) after data scientists completed the predictions for Cohort 2, the PI released the outcome data for this cohort and final diagnostic accuracy measures were computed then.

Reporting for specific materials, systems and methods

We require information from authors about some types of materials, experimental systems and methods used in many studies. Here, indicate whether each material, system or method listed is relevant to your study. If you are not sure if a list item applies to your research, read the appropriate section before selecting a response.

Materials & experimental systems

n/a	Involved in the study	
☒	☐	Antibodies
☒	☐	Eukaryotic cell lines
☒	☐	Palaeontology
☒	☐	Animals and other organisms
☐	☒	Human research participants
☒	☐	Clinical data

Methods

n/a	Involved in the study	
☒	☐	ChIP-seq
☒	☐	Flow cytometry
☒	☐	MRI-based neuroimaging

Human research participants

Policy information about studies involving human research participants

Population characteristics	The current data set consisted of 750 patients (aged 59 ± 17 years, 42% female) from the first EMPIRE cohort and 500 patients (aged 59 ± 16 years, 49% female) from the second EMPIRE cohort.
Recruitment	This prospective observational cohort study recruited consecutive, non-traumatic chest pain patients transported by emergency medical services to one of three UPMC-affiliated tertiary care hospitals (UPMC Presbyterian, Mercy, and Shadyside). As per prehospital medical protocols, standard 10-second 12-lead ECGs are obtained on all patients with suspected ACS during first medical contact. If the initial patient evaluation by paramedics was judged to be highly suspicious for cardiac ischemia, then the ECG was transmitted to UPMC medical command center, where the raw digital ECG data are permanently stored. In the EMPIRE study, we recruited all consecutive chest pain patients with transmitted ECG data and followed them up for 30 days from the indexed encounter. This study is not a clinical trial and therefore was not registered with ClinicalTrials.gov
Ethics oversight	This study was approved by the Institutional Review Board of University of Pittsburgh under a waiver of informed consent.

Note that full information on the approval of the study protocol must also be provided in the manuscript.
